# BARRIERS AND FACILITATORS TO CARE PEOPLE WITH COGNITIVE IMPAIRMENT IN HOSPITALS OF CHINA: A QUALITATIVE STUDY

**DOI:** 10.1093/geroni/igae098.3307

**Published:** 2024-12-31

**Authors:** Yalin Lv, Yinuo Ou, Zhijiang Li, Jie Jia, Juan Li

**Affiliations:** Huashan Hospital Fudan University, Shanghai, Shanghai, China (People’s Republic); Huashan Hospital Fudan University, National Center for Neurological Disorders of China, Shanghai, Shanghai, China (People’s Republic); Huashan Hospital Fudan University, Shanghai, Shanghai, China (People’s Republic); Huashan Hospital Fudan University, Shanghai, Shanghai, China (People’s Republic); Huashan Hospital Fudan University, Shanghai, Shanghai, China (People’s Republic)

## Abstract

Cognitive impairment refers to various degrees of cognitive decline in memory, attention, language, execution, and orientation, significantly impacting people’s daily activities and quality of life. With the aggravation of aging population, China has a huge number of people with cognitive impairment and high demand for care. Although there are several international guidelines of care individuals with cognitive impairment, class A tertiary hospitals in China have not well applied them to the clinical care. This study aimed to understand the context of care people with cognitive impairment in hospitals and to explore potential barriers and facilitators to evidence-informed practices. Semi-structured interviews were conducted with nurses from various departments in a Class A tertiary hospital in China. We developed an interview guide based on the Theoretical Domain Framework and guidelines. Data were analyzed using a framework analysis and domains were identified as salient based on their frequency. Finally, 11 domains related to the clinical care of people with cognitive impairment as well as 6 “barrier” and 11” facilitator “ domains were identified. The “barrier” domains were social influences, environmental context and resources, social/ professional role and identity, beliefs about consequences, beliefs about capabilities and intentions. The “facilitator” domains were knowledge, physical skills, cognitive, interpersonal skills, social influences, environmental context and resources, social/ professional role and identity, behavioral regulation, memory attention and decision processes, beliefs about consequences, beliefs about capabilities and intentions. These will inform collaboration of multiple disciplines and evidence-based intervention to improve the practice of caring cognitive impairment individuals in hospitals.

